# UniMoG—a unifying framework for genomic distance calculation and sorting based on DCJ

**DOI:** 10.1093/bioinformatics/bts440

**Published:** 2012-07-18

**Authors:** Rolf Hilker, Corinna Sickinger, Christian N.S. Pedersen, Jens Stoye

**Affiliations:** ^1^Computational Genomics, Faculty of Technology, Bielefeld University, 33615 Bielefeld, Germany, ^2^Institute for Bioinformatics, Center for Biotechnology (CeBiTec), Bielefeld University, 33615 Bielefeld, Germany, ^3^Bioinformatics Research Center (BiRC), Aarhus University, 8000 Aarhus, Denmark and ^4^Genome Informatics, Faculty of Technology, Bielefeld University, 33615 Bielefeld, Germany

## Abstract

**Summary:** UniMoG is a software combining five genome rearrangement models: double cut and join (DCJ), restricted DCJ, Hannenhalli and Pevzner (HP), inversion and translocation. It can compute the pairwise genomic distances and a corresponding optimal sorting scenario for an arbitrary number of genomes. All five models can be unified through the DCJ model, thus the implementation is based on DCJ and, where reasonable, uses the most efficient existing algorithms for each distance and sorting problem. Both textual and graphical output is possible for visualizing the operations.

**Availability and implementation:** The software is available through the Bielefeld University Bioinformatics Web Server at http://bibiserv.techfak.uni-bielefeld.de/dcj with instructions and example data.

**Contact:** rhilker@cebitec.uni-bielefeld.de

## 1 INTRODUCTION

Genome rearrangements describe the dynamics of evolution at an abstracted genomic level, in contrast to local mutations of single DNA base pairs. Very little is known about the exact procedure of rearrangement events and how and when they are triggered. More detailed knowledge of evolution could help to improve the understanding of the mechanisms important for survival and development of species. The evolutionary distance between at least two organisms with shared gene content can be estimated by solving the combinatorial problem of finding a possible sequence of rearrangement operations among their shared genes under the aspect of parsimony. Thus, all genes unique to one of the genomes are ignored and only one representative among duplicated genes is chosen for the comparison.

In recent years, large amounts of genomic data have become available and genome comparison has become a routine task. For example, the Chimpanzee Sequencing and Analysis Consortium (2005) compared chimpanzee and human genomes and developed a catalogue of genetic differences. Since both are closely related, only one fusion of two chromosomes and several inversions were identified. Another example is the comparison of human and mouse genomes by Pevzner and Tesler (2003). Among other methods they used GRIMM (Tesler, 2002b) for the analysis, because utilizing automated methods allows for easier and faster analyses, no matter how divergent the investigated organisms are. GRIMM is based on the Hannenhalli and Pevzner (HP) model (Hannenhalli and Pevzner, 1995), thus its set of rearrangement operations comprises inversions, translocations, fusions and fissions of linear genomes. However, one can investigate the phylogenetic distance under different aspects and the HP model is only one of the common models. Besides the HP model we consider four additional models. The inversion model (Hannenhalli and Pevzner, 1999) allows for inversions of internal genomic regions in linear, uni-chromosomal genomes, while the translocation model (Hannenhalli, 1996) comprises the exchange of two linear chromosome ends. As already mentioned, HP combines both models and adds fusions and fissions of two chromosomes to the repertoire of rearrangement operations. Among the included models, the most general is the double cut and join (DCJ) model (Bergeron *et al.* 2006b; Yancopoulos *et al.* 2005), which allows for all common rearrangement operations: inversions, translocations, fusions, fissions, circularizations and decircularizations. Besides these operations, block interchanges, which describe the exchange of two DNA segments, can be mimicked through two operations by all models, except the inversion model. Finally, the restricted DCJ model (Kováč *et al.*, 2011) allows the same operations as the DCJ model, but constricts it by requiring immediate decircularization in the next step for emerging circular chromosomes.

**Fig. 1:**
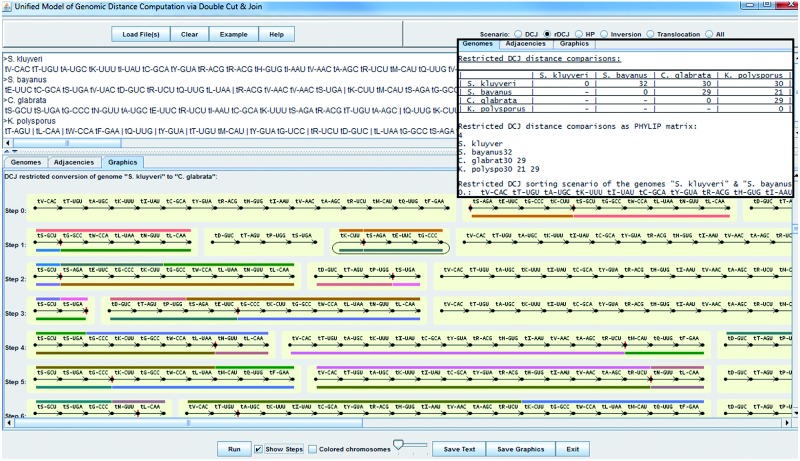
Two of the three output levels of a restricted DCJ sorting scenario involving the common t-RNA genes of four yeast genomes. The circular chromosome in step one is directly reincorporated in the next step according to the restricted DCJ definition

In our software, UniMoG, the DCJ Adjacency Graph data structure (Bergeron *et al.*, 2006b), serves as basis for all calculations, and in contrast to GRIMM it implements, based on DCJ, all of these five distance models and is able to return either the desired distance or the distance and a corresponding optimal sorting scenario. For fast comparisons between the different distances, it is also possible to calculate all five distances and sorting scenarios at once, if applicable. Another advantage is that the input is neither limited to two genomes at a time nor can genes only be represented by integers. Instead, gene names are converted to integers for the internal representation. In the case of multiple input genomes, all of them are compared pairwise with each other. The distance results are then returned in a matrix, which is also provided in PHYLIP format (Fig. 1, inset), and can further be fed into distance-based phylogenetic tree reconstruction methods, possibly after applying distance correction models like the ones presented by Lin and Moret (2008).

UniMoG was implemented with a strong focus on computational efficiency. Therefore, all five distance calculations and the DCJ sorting are carried out in linear time as explained in Bergeron *et al.* (2006b), Erdős *et al.* (2011), Pevzner and Tesler (2003) and Tesler (2002a). For restricted DCJ sorting, we implemented the linearithmic time algorithm of Kováč *et al.* (2011). The implemented translocation sorting algorithm, explained in Bergeron *et al.* (2006a), was chosen even though its worst case running time is cubic, because in practice it almost always runs in linear time. Our implementation of the inversion sorting algorithm is the sequence augmentation algorithm introduced by Tannier *et al.* (2007) with a quadratic worst case running time, based on the data structures from Bergeron *et al.* (2005). This algorithm also defines the running time of the HP sorting algorithm, since it uses the preprocessing explicated in Tesler (2002a) and afterwards hands over the concatenated genomes to the inversion sorting algorithm. Although GRIMM still contains an error, revealed by Jean and Nikolski (2007), we use their corrected capping and concatenation algorithm. Note that all of these algorithms return only one of possibly many sorting scenarios. Sampling uniformly among all scenarios will be subject of a future version of UniMoG.

Because of the efficient implementation, UniMoG can handle large genomes and was tested with genomes up to 32 500 genes without encountering any problems. For further improvement of the computational performance, regions with identical gene order can be merged into larger synteny blocks, since none of the considered models can break up conserved blocks.

For an intuitive handling, the output of UniMoG is divided into three levels (Fig. 1): first, the graphical output is designed for closely studying the rearrangement scenarios, highlighting each performed operation and allowing three different zoom levels. Furthermore, when color mode is active, each chromosome is assigned a unique color for easier analysis of large genomes. Second, an optimal sorting scenario is returned in text format, which allows for easy reuse of intermediate genomes. Finally, the results are also returned as a list of adjacencies of each intermediate genome. The integrated save functions allow quick saving of graphical or textual output data.

*Conflict of Interest*: none declared.
